# Potential Diagnostic and Prognostic Biomarkers of Epigenetic Drift within the Cardiovascular Compartment

**DOI:** 10.1155/2016/2465763

**Published:** 2016-01-28

**Authors:** Robert G. Wallace, Laura C. Twomey, Marc-Antoine Custaud, Niall Moyna, Philip M. Cummins, Marco Mangone, Ronan P. Murphy

**Affiliations:** ^1^School of Health & Human Performance, Dublin City University, Ireland; ^2^INSERM 1083, Angers Medical University, France; ^3^School of Biotechnology, Dublin City University, Ireland; ^4^The Biodesign Institute, School of Life Sciences, Arizona State University, USA

## Abstract

Biomarkers encompass a wide range of different measurable indicators, representing a tangible link to physiological changes occurring within the body. Accessibility, sensitivity, and specificity are significant factors in biomarker suitability. New biomarkers continue to be discovered, and questions over appropriate selection and assessment of their usefulness remain. If traditional markers of inflammation are not sufficiently robust in their specificity, then perhaps alternative means of detection may provide more information. Epigenetic drift (epigenetic modifications as they occur as a direct function with age), and its ancillary elements, including platelets, secreted microvesicles (MVs), and microRNA (miRNA), may hold enormous predictive potential. The majority of epigenetic drift observed in blood is independent of variations in blood cell composition, addressing concerns affecting traditional blood-based biomarker efficacy. MVs are found in plasma and other biological fluids in healthy individuals. Altered MV/miRNA profiles may also be found in individuals with various diseases. Platelets are also highly reflective of physiological and lifestyle changes, making them extremely sensitive biomarkers of human health. Platelets release increased levels of MVs in response to various stimuli and under a plethora of disease states, which demonstrate a functional effect on other cell types.

## 1. Introduction

Biomarkers encompass a wide range of different measurable indicators, including protein, polysaccharides, and nucleic acids, and are distinct, both quantitatively and qualitatively, from common circulating biomolecules, such as albumin. They represent a tangible link to physiological changes occurring within the body and ideally enable diagnostic and/or prognostic evaluations to be determined with respect to disease. Accessibility, sensitivity, and specificity are significant factors in biomarker suitability. The initiation and progression of atherosclerosis are traditionally measured by internal intima-medial thickness (IMT) [1]. However, detection of inflammatory processes through use of biomarkers may facilitate earlier diagnosis well before the clinical threshold and enable preventive remedial action to be taken. A number of commonly used indicators exist, and their wide use in the clinical setting reported in literature indicates large consensus of their efficacy. Nevertheless, new biomarkers continue to be discovered, and questions over appropriate biomarker selection and assessment of their usefulness as reliable indicators of disease remain. In this context, the emerging field of epigenetics has enormous potential as biomarkers of cardiovascular risk. By analysing environmentally induced changes in chromosomes and monitoring these alterations over time, determination of the epigenetic “drift” is possible. Such data have the potential to provide novel therapeutic targets for vascular-associated chronic illnesses and initiate novel drug-development pipelines.

## 2. CVD and Problems Posed

Cardiovascular disease (CVD) is an umbrella term for a class of diseases involving the cardiovascular system and consequently includes heart failure, arrhythmia, angina, hypertension, high cholesterol, and stroke amongst others. Vascular inflammation is a key contributor to CVD etiology, with inflammation and CVD risk factors closely interrelated. CVD risk factors, such as hypertension, and hypercholesterolemia are associated with higher circulating concentrations of inflammatory biomarkers [2]. Types of CVD are varied and complex; hence there are multiple pathological mechanisms through which disease may arise. Atherosclerosis is the most common of the vascular inflammatory diseases. It is defined as chronic, specifically affecting arterial blood vessels, and caused by dynamic dysfunction of the endothelium. Endothelial dysfunction is regarded as the earliest detectable indicator of CVD and is used as an independent predictor of future disease occurrence [3].

Dysregulation of endothelial homeostasis is characterised by reduced availability of nitric oxide and is an acknowledged marker of early atherogenesis [4] and a multistage process. Following injury, the resulting immune response leads to inflammation at the site. Macrophage cells are typically the first type to respond. They act by secreting proteins (cytokines and chemokines) in order to recruit additional immune cells to the site of injury [5]. Vascular Cellular Adhesion Molecule 1 (VCAM1) and Intracellular Adhesion Molecule 1 (ICAM1) are other important mediators in cell-cell and cell-matrix interactions during inflammation and resultant aberrant immune response. As such, they may all act as “biomarkers” of inflammation. However, their use is relatively broad.

Biomarkers of associated inflammatory responses yield an alternative/indirect means of detection. Oxidative stress, for example, is widely associated with atherosclerosis, myocardial infarction, and so forth and is known to play a critical role in the genesis and continuance of inflammation through interruption of normal cell signaling mechanisms [6]. As such, a host of related biomarkers have been employed, including high-sensitivity C-reactive protein (C-RP) and interleukin-1, interleukin-6, and interleukin-10 [7, 8].

A recently published longitudinal study conducted over a twenty-year period investigated the relationship between subclinical atherosclerosis and a host of biomarkers including cytokines/adipokines, thrombosis, and adhesion molecules. IMT measurement was used as control and conducted over four time-points during the investigative period. The test group consisted of 886 individuals with type 1 diabetes. Logistic regression models carried out by the team suggest that individual biomarkers were not predictive of/associated with subclinical atherosclerosis. However, composite scores of acute-phase reactants, cytokines/adipokines, and thrombolytic factors were nevertheless associated with higher levels of atherosclerosis at Epidemiology of Diabetes Interventions and Complications (EDIC) year 12 [1].

## 3. Predictor Potential versus Risk Assessment and Robustness

While biomarkers of other diseases may be derived from surgical biopsy, in terms of detecting vasculature-derived disease, blood represents perhaps the simplest and most logical of sources from which to obtain biological markers. Analysis of blood and bodily fluids in general has emerged as a form of “liquid biopsy.” This has primarily been used in the field of cancer diagnosis but equally holds promise for diagnosis of other conditions.

One of the major difficulties in identifying suitable biomarkers is determining those which independently signal a change within the system, without becoming overwhelmed by the myriad of processes surrounding them. Sensitivity and specificity are paramount [9]. Numerous signalling molecules, such as inflammatory cytokines, are proposed as important factors related to atherosclerotic pathogenesis. Tumor necrosis factor alpha (TNF*α*), for example, is widely used as a marker of vascular inflammation [10, 11]. Cung et al. [12] acknowledge that it is readily capable of activating endothelial cells (ECs). However, they state that, due to the presence of thousands of other factors, the discrete assessment of individual cytokines, such as TNF*α* or CD40, may fail to capture the global inflammatory stimulus that the blood conveys to the endothelium. As such, they believe that the complex composition of blood results in a tangible gap between biomarker and clinical outcome. A similar sentiment is shared in another recent publication, where the presence of interferons due to the presence of disease inhibited biomarker interpretation [13]. This conflicts with the use of nuclear interferon-inducible-16 (IFI16), which acts as DNA sensor in inflammasome signalling, and may also function as a suitable inflammatory biomarker [14]. It has previously been used as a biomarker of cognitive dysfunction [15]. Nevertheless, this is a serious challenge to the usefulness of blood-based biomarkers [12]. If traditional markers of inflammation are not sufficiently robust in their specificity, then perhaps alternative means of detection may provide more information.

Data from studies carried out by Brindle et al. [16] has shown that traditional factors, such as smoking and diet, lack accuracy and may even result in overestimation of disease risk in low-risk populations and* vice versa*. Involvement of hereditary factors may represent a more significant agent in both vascular remodelling and disease development. Indeed, with regard to coronary artery disease (CAD), genome-wide association studies have led to the identification of a large number of single nucleotide polymorphisms (SNPs) associated with increased risk of developing the disease [17]. However, each of these approaches is based on the fundamental principle that genetic determinants lie within the static DNA code of individuals; the hypothesis being that a person may, in effect, be more susceptible to succumbing to disease based on their unique genetic profile. Nevertheless, the genetic code of individuals has long been known to be flexible and adaptable. Such adaptations may occur in the long term through evolution or may occur as a more direct reaction to various external stimuli by means of up/downregulation of genes in order to best meet the adaptive demands of certain situations.

## 4. Epigenetics, Aging, and Chronic Disease

Biomarkers discussed thus far are predominantly diagnostic in nature. However, the emerging field of epigenetics may hold enormous predictive potential. Owing to the existence of numerous conflicting interpretations of what the term meant, epigenetics was finally defined in 2008 as “a stably heritable phenotype resulting from changes in a chromosome without alterations in the DNA sequence” [18]. DNA methylation, the covalent addition/removal of methyl groups, is an example of such independent modification. Histone protein modification is an area of some debate and had been considered not to be epigenetically regulated [19], though current thinking places it firmly within the epigenetic realm [20]. More recently, noncoding RNA (ncRNA) has also been included as major players in the epigenetic domain, with microRNA (miRNA) recognised as having an important regulatory function. Importantly, epigenetic modifications persist within the cell [19].

Following methylation and/or histone N-terminal modification (acetylation, deacetylation, phosphorylation, and ubiquitylation), the epigenome is altered. Effect on gene transcription is nuanced and may vary depending on context. Regulation of gene expression was originally believed to occur through alteration of transcriptional start sites (TSSs). Methylated regions have been linked with particular chromatin features associated with transcriptional repression, especially H3K9me3 and methyl-CpG-binding protein 2 (MECP2). Methylation in other regions of the genome is increasingly accepted as having functional relevance and presents fundamentally more dynamic patterns of methylation than those discovered in TSSs [21]. There, for example, methyl-binding domain (MBD) proteins may serve to coalesce DNA, which, as with TSSs, then recruit histone deacetylases and effectively silence that sequence. Current research indicates that CpG methylation is notably avoided at sites close to TSSs. It has been proposed that those TSSs that are methylated exhibit long-term silencing [21].

**Figure 1:**
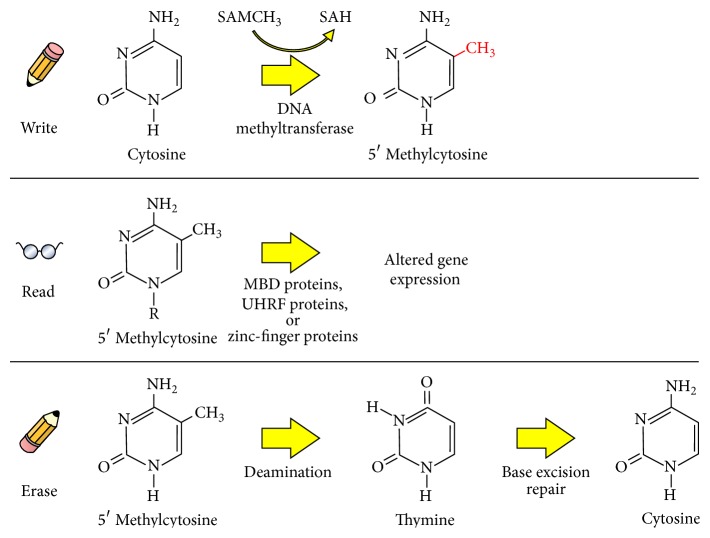
DNA methylation (writing, reading, and erasing) represents one of a number of means through which epigenetic modification of DNA may occur. Methyltransferase enzymes serve to methylate cytosine residues, particularly in CpG islands. Methyl-CpG-binding domain (MBD) proteins, Ubiquitin-like, containing PHD and RING finger domain (UHRF) proteins or zinc-finger proteins may each play a role in reading methylated DNA. Deamination of 5′ methylcytosines to form thymine residues that are subsequently removed via the base excision repair mechanism is one way that methylated bases may be restored.

In contrast to silencing, transcriptional stimulation may instead occur as a result of methylation. This has been observed largely in regions located away from TSSs, a phenomenon termed the “DNA methylation paradox” [22]. Furthermore, over 50% of vertebrate genes contain short (~1 kb) CpG-rich sequences, with the remaining genome deplete of such regions. In mammalian cells, methylation generally occurs within CpG dinucleotides, typically in cytosine residues that are followed by guanine. Hence, development of detailed epigenome maps in normal and disease states (and thus understanding the distribution of methylation across the genome) is requisite to understanding the function of DNA methylation [21]. The methylation mechanism is highly regulated and, importantly, is a fluid, dynamic, and reversible process, orchestrated through a complex network of epigenetic writer, reader, and erasers (see Figure 1).

**Figure 2:**
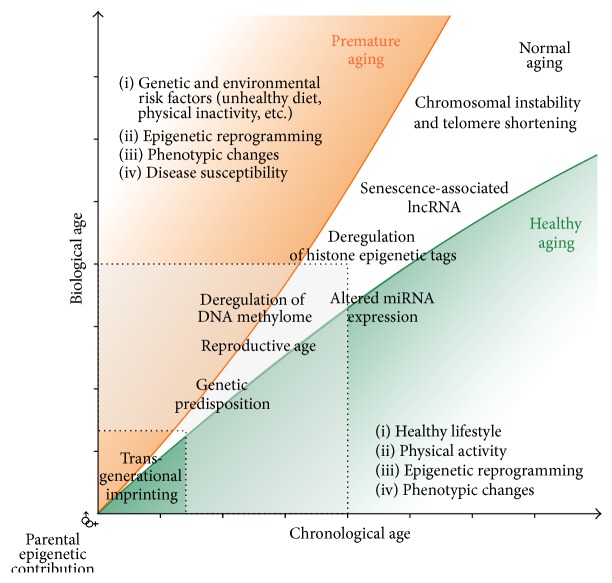
Epigenetic drift over time can result in measurable differences between biological and chronological age. Epigenetic changes have been found to be reflective of lifestyle and may act as functional biomarkers of disease before clinical threshold is reached.

Epigenetic drift is the term given to epigenetic modifications as they occur as a direct function with age. While age is a known risk factor for many diseases, age-related methylation has been found to occur differentially at specific sites along the genome. Hence, investigation of epigenetic drift may result in the discovery of key underlying biomarkers of numerous age-related pathologies [23]. Research has shown that tissue-specific age-associated CGs are commonly found outside CpG islands with decreased methylation. Such sites have been linked with higher tissue-specific expression levels compared to those with increased methylation [24, 25]. Some debate has arisen as to the complexity of the methylation mechanism. Recent studies by Valencia-Morales Mdel et al. [25] favour a common instructive mechanism. However, Day et al. [24] believe a much more complex mechanism than a simple epigenetic drift model is at play. The later theory would suggest that epigenetic methylation could potentially represent a viable type of biomarker. Indeed, DNA methylation-derived measures of aging have been found to predict mortality independently of health status and other known genetic factors [26]. The aging effects of epigenetic drift over time are highlighted in Figure 2.

Physical activity (PA) is a modifiable lifestyle choice, with enormous health and fitness benefits. Regular PA is a known requirement to maintain a healthy cardiovascular system [27] and has been shown to produce a long-term anti-inflammatory effect in vasculature [28]. Recent efforts have been made to assess the effects of exercise as an epigenator in DNA from a variety of tissues. Human skeletal muscle has been found to be influenced by exercise applied as a physiological stressor. Genes previously found to be differentially methylated in type 2 diabetes (PGC-1*α*, PPAR-*δ*, TFAM, citrate synthase, and PDK4) were evaluated, and methylation was found to be lower following acute exercise. Muscle-specific transcription factors remained unchanged [29].

Rather than taking baseline single point blood sample measurements, stressing the cardiovascular system (*in vivo* instead of* in vitro* stress models) by prescribed exhaustive exercise or by the use of acute models of physical inactivity may provide much more valuable and conclusive evidence in relation to the body's response to stress. Among the first to employ global DNA methylation pattern analysis was a study investigating the effects of a six-month exercise intervention on DNA methylation in adipose tissue of healthy men. Results demonstrated genome-wide DNA methylation changes, and 17,975 individual CpG sites exhibited altered levels of DNA methylation in response to exercise [30]. Conversely, a separate study by Horvath et al. [31] in adipose tissue using the same methylation change data as a comparator found that such short-term weight loss did not impact the DNA methylation age of the tissue.

Obesity, often associated with sedentary lifestyle, has been demonstrated in another study as an accelerant of epigenetic change. Methylation was found to occur in DNA from liver tissue in direct correlation with body mass index (BMI) [32]. This corresponds with evidence uncovered by Horvath et al. [31], in a comparable study where the relationship between DNA methylation and BMI in a variety of tissues was examined, including liver. The authors were able to discern and validate age acceleration in this tissue. Importantly, results highlighted tissue-specific changes, indicating that individual CpG sites may be unsuitable for global comparisons between differing tissue types. Instead, an aggregate approach, validated across cells, complex tissues, and organs, was proposed and used in the analysis. Furthermore, the authors call for caution in the use of BMI as a measure of adiposity, instead, citing muscle mass in association with methylation as a more suitable measure of epigenetic aging for this type of research.

## 5. Arterial Remodelling and Blood

Blood vessels are readily capable of remodelling/altering themselves in response to hemodynamic prompts associated with variations in blood flow. Significant variations of flow pattern occur, such as that produced in response to exercise or sedentary behaviour. In some cases, these may be pathological flow types that lead to aberrant remodelling and development of disease status. Calls were recently made for deeper investigation into possible correlation between sedentary behaviour and vascular function, particularly in children [3]. A number of studies used the example of the relationship between sedentary television viewing time and cardiovascular risk. Gabel et al. [33] investigated the relationship between sedentary television viewing time in children (aged 7–10 years) and inflammatory and endothelial function biomarker levels. Here, the team found that following adjustment for age, weight, sex, and so forth, each additional hour per week of television viewing was associated with 4.4% (95% CI: 2.1, 6.7) greater C-RP and 0.6% (0.2, 1.0) greater VCAM-1. The authors advocate a more rigid and longer study to fully assess this association. Nevertheless, the relationship between sedentary lifestyle and inflammation was clearly drawn. As such, any further investigation could potentially involve epigenetic markers.

The complex composition of blood as a challenge to biomarker efficacy, discussed previously, was examined in a recent publication by Yuan et al. [23], where whole blood tissue was analysed for epigenetic drift. Key amongst the findings was the fact that the majority (80%) of epigenetic drift observed in blood was independent of variations in blood cell composition. When adjusted for composition, results demonstrated a reduction in epigenetic drift attributable to the increase in the granulocyte : lymphocyte ratio, with a concomitant enrichment of age-hypermethylated CpG islands. This is a significant boost for the use of epigenetic biomarkers, where traditional types have been found to succumb to sensitivity and specificity issues.

## 6. Circulating Biomarkers: Microvesicles, miRNA, and Platelets

There can be no doubt that markers of endothelial dysfunction and epigenetic screening may be of prognostic value in detection of disease. In the immediate future, however, microvesicles (MVs) may represent a more feasible opportunity to translate potential prognostic biomarkers to clinical practice. These secreted vesicles permit reprogramming of recipient cells and are easily obtained in bodily fluids [34].

Different types of MVs are released by mammalian cells into the extracellular space, including blood. Critically, they carry membrane and cytosolic components [35] and maintain both a similar topology and antigenic signature to the parent/donor cell and yet may be extracted from sites distal to the parent cell within the body via blood. MVs are found in plasma and other biological fluids in healthy individuals. Altered MV profiles may also be found in individuals with various diseases [36]. They may be released under a variety of different circumstances, for example, when cells undergo the natural processes of apoptosis or necrosis or when injured. MVs may be divided into one of the three categories, outlined in Table 1.

Due to their origin from different cell types, their differential mechanism of biogenesis, and resultant variable composition, MV effector function is believed to be similarly varied. As such, it is believed that microvesicles represent detectable units involved in intercellular exchange. Indeed, particular biomarkers have been associated with particular types of vesicles; for example, the presence of CD80 and CD86 is indicative that exosomes found in plasma may be of antigen presenting cell (APC) origin [37]. In terms of the vasculature, endothelial cell-derived microparticles (MPs), therefore, display characteristic antigen markers that allow for their identification and detection. An interesting aspect of MP surface marker research is the requirement of specific techniques to generate MPs that carry specific markers. For example, work carried out by Takahashi et al. [38] included generation of aortic EC-derived MPs bearing VE-cadherin in response to TNF*α* stimulation. Neither hydrogen peroxide (H_2_O_2_) nor cigarette smoke extract (as the study also looked at pulmonary vascular ECs) treatment of the same cell type yielded a similar result. Furthermore, the relationship between expression levels of particular antigens and levels detectable on MPs did not appear to directly correlate.

Examinations on circulating endothelial- and platelet-derived MPs have been conducted. Findings suggest that levels of circulating endothelial and platelet MPs correlate to the size of myocardium at risk in patients with ST-elevation myocardial infarction. This is indicative of the fact that they reflect the severity of endothelial injury and platelet activation during myocardial ischemia [39].

As with cytokines, discussed previously, the sheer number of MVs present in vasculature from all cell types may present an overarching masking effect that renders individual effects difficult to perceive. Of interest is research carried out by Garnacho-Montero [40] on the reliability of circulating cell-free DNA (cf-DNA) concentrations, compared to C-RP, procalcitonin (PCT), and eosinophil count, in infection diagnosis in patients with systemic inflammatory response syndrome. Results showed that cf-DNA levels did not correlate with C-RP or PCT in septic patients. Those with acute myocardial infarction (known to increase cf-DNA levels) were excluded from the study. No research has so far been conducted with regard to the reliability of cf-DNA as an indicator of vascular inflammation.

## 7. MicroRNA

Perhaps one of the most important components of MPs is miRNA. These are short noncoding RNA molecules, approximately 22–25 nucleotides in length [41], the actual length of which is defined by the specific argonaute involved in its genesis [42]. Since their discovery in 1993, during experimentation on the nematode,* Caenorhabditis elegans* [43], the field of research has expanded to encompass genome-wide studies. Current opinion hails miRNA as molecules of vast regulatory potential [44]. Target identification/characterisation remains a significant challenge, given the vast numbers of miRNAs so far discovered.

Nevertheless, a number of potential circulatory miRNA markers have been determined. Of those investigated thus far, mir-155 has been revealed as an important herald of inflammatory response. Analysis of both* in vitro* and* in vivo* models has revealed mir-155 to be involved in a negative feedback loop. Impeding mir-155 resulted in downregulation of inflammatory cytokines, IL-6 and TNF*α*. Absence of these cytokines served to impede atherosclerotic development. Significantly, mir-155 is postulated to be responsible for posttranslational regulation of the MAPK pathway by targeting MAP3K 10 [45].

## 8. Platelets Act as Biomarkers

Circulating blood platelets are 2–4 *μ*m sized anucleate fragments of precursor cells called megakaryocytes. Their production is arguably the most elegant and distinct developmental process in eukaryotes [46]. An obvious advantage of using platelets as biomarkers of human health and disease is their fast, simple, and minimally invasive accessibility from whole blood in large numbers, representing the second most abundant cell type in blood. Platelets are also highly reflective of physiological and lifestyle changes, making them extremely sensitive biomarkers of human health. Although anucleate, platelets retain a sophisticated repertoire of messenger RNA, miRNA, and protein which contribute to primary (adhesion, activation, secretion, and aggregation) and alternative (immune regulation, inflammation, RNA transfer, and tumour metastasis) functions [47].

Typical biomarkers of platelet health include identification of primary platelet function changes in activation, aggregation, and secretion. A multitude of techniques, most notably flow cytometry (to measure glycoprotein activation-dependent changes, exposure of granule membrane proteins, platelet-leukocyte aggregate, etc.), aggregometry, and Enzyme-Linked Immunosorbent Assay (ELISA) have previously been used to identify these changes. Lack of standardisation between labs and preactivation of platelets due to methodological pitfalls are major problems when analysing platelet-derived biomarkers, warranting a pressing requirement for reliable platelet biomarkers [48].

## 9. Platelet miRNA as Epigenetic Biomarkers

It has been well documented that activated platelets also release the most abundant cell-derived MVs, accounting for 70–90% of circulating peripheral MVs. These MVs harbour an array of cell components including protein, DNA, and RNA and can act as a network of communication by selectively interacting with target cells. Platelets release increased levels of MVs in response to various stimuli and under a plethora of disease states including CVD, cancer, and neurodegenerative diseases.

## 10. Platelet miRNA Has a Functional Effect on Other Cells

The modern “omics” revolution enables simultaneous quantification of hundreds of molecules from a single sample and may be more reflective of platelet function changes, providing novel biomarkers for a range of diseases [47]. Platelets provide considerable contribution to the circulating miRNA pool and their harboured miRNAs have been labelled as markers of mature megakaryocyte miRNA [49]. As platelets are anucleate and have a limited lifespan, platelet-derived miRNA could represent an ideal marker of temporal and modifiable epigenetic drift [48].

**Figure 3:**
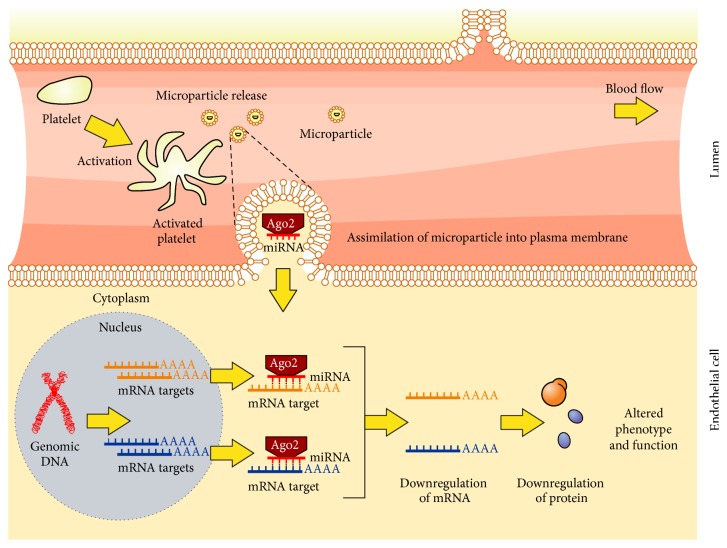
Platelet MVs act as intercellular transporters of functional Ago2-miRNA complexes. Activated platelets release microvesicles packaged with functional Argonaute 2- (Ago2-) miRNA complexes which can be assimilated by human aortic endothelial cells (HAECs). Platelet-derived miRNA accumulates within the cell, regulating expression of endothelial genes at the mRNA and protein level, resulting in altered phenotype and function.

Furthermore, studies by Laffont et al. [50] and Gidlöf et al. [51] demonstrate the functionality of platelet miRNA, whereby functional complexes of miR-223 and Argonaute 2 protein (Ago2) packaged in MVs from activated platelets could modulate expression of targeted endothelial cell mRNA transcripts FBXW7 and EFNA1. This same complex has also been shown to reduce expression levels of another transcript, the insulin-like growth factor 1 receptor in endothelial cells, and promote human umbilical vein endothelial cell (HUVEC) apoptosis [52]. This type of platelet MV-cell interaction is illustrated in Figure 3.

## 11. Platelet miRNA as a Biomarker of CVD

Platelets play a key role in CVD progression where the normal platelet response is modified by enhanced proaggregatory processes and decreased antiaggregatory mechanisms thereby generating a condition of platelet hyperaggregability on an acute and chronic level. Following atherosclerotic plaque rupture in severe CVD states, platelets adhere to the disrupted plaque and aggregate to form a prothrombotic surface, subsequently encouraging thrombosis and vascular occlusion. Platelet miRNAs have been differentially implicated in the presence and progression of a range of diseases, including but not limited to various CVDs such as Acute Coronary Syndrome (ACS), Atrial Fibrillation (AF), and coronary artery disease (CAD) [53].

Platelet miRNA profiles are differentially expressed in ACS. In a study by Ward et al. [54], blood was obtained from 13 patients with ACS presenting with either ST Segment Elevation Myocardial Infarction (STEMI) or Non-ST Segment Elevation Myocardial Infarction (NSTEMI). In STEMI, the artery is usually completely occluded as compared to NSTEMI, which displays partial occlusion [55]. Four platelet enriched miRNAs were differentially expressed between groups. Platelet enriched miR-186-5p and miR-342-3p were significantly lower in patients with STEMI as compared with NSTEMI. In contrast, platelet enriched miR-25-3p and miR-221-3p were significantly higher in patients with STEMI as compared with NSTEMI.

Circulating levels of the platelet enriched miR-328 have also been linked to AF in 2445 offspring in the Framingham Heart Study [53]. This miR controls particular genes involved in inflammation, vascular function (ABCG2), cell signalling, and cellular aging (H2AFX), which constitute some of the mechanisms in the development of AF. miR-328 expression was decreased in subjects with AF in comparison to subjects without AF. Furthermore, in a separate investigation, Sondermeijer et al. [56] demonstrated by microarray analysis that platelet-derived miRNAs (miR-624 and miR-340) were significantly elevated in CAD patients compared to healthy individuals.

## 12. Platelet Mitochondria as a Biomarker

Investigation of metabolic function in platelets for disease diagnosis and prognosis is a new area of translational research [57]. Platelets contain functional mitochondria whose activity is modulated in different pathological states such as CVD [58]. This suggests that platelets can sense metabolic stress and act as surrogate markers of mitochondrial dysfunction in remote inaccessible tissue types [59].

The traditional role of mitochondria in the platelet was considered to supply energy in the form of ATP for primary platelet functions: adhesion, activation, and aggregation. However, novel functions for mitochondria continue to emerge. Mitochondrial membrane potential (ΔΨ_m_) is reduced in a subtype of platelets known as Collagen and Thrombin Activated (COAT) platelets. Dual activation of platelets with collagen and thrombin results in striking alterations in function and structure. COAT platelets differ from typical “activated platelets” by exhibiting a myriad of features such as phosphatidylserine exposure due to cytoskeletal reorganisation, high microparticle release, and increased levels of fibrinogen on the platelet surface. In addition, mitochondria have been noted to be involved in ATP-controlled thrombotic signalling and platelet apoptosis [60].

## 13. Platelet mtDNA Methylation

Platelets have a higher degree of ATP turnover than resting mammalian muscle which holds high levels of mitochondria, suggesting a critical role for mitochondria in platelet function and huge requirement for energy during platelet activation. As with nuclear DNA, mitochondrial DNA (mtDNA) has the potential to be methylated by factors such as disease, ageing, and environmental exposure, moderating control of mitochondrial gene expression. In tandem with platelet miRNA biomarkers, simultaneous understanding of epigenetic regulation of mitochondrial genes in platelets is proving crucial to understanding their implication in CVD development. Recently, Baccarelli and Byun [61] investigated platelet mtDNA methylation in a selection of mitochondrial genes including NADH dehydrogenase (*MT-MD5*), cytochrome c oxidase (MT-CO1, MT-C02, and MT-C03), tRNA leucine 1 (MT-TL1), and ATP synthase (MT-ATP6 and MT-ATP8) amongst CVD patients and healthy individuals by bisulfite PCR coupled with pyrosequencing. CVD patients had significantly higher mtDNA methylation than healthy individuals in MT-CO1, MT-C02, MT-CO3, and MT-TL1, genes involved in ATP synthesis.

DNA methylation in platelet mitochondria could be a potential contributor to CVD development through regulation of platelet function. The role of mtDNA methylation in platelet function has yet to be fully determined, but this study is the first to portray platelet mtDNA methylation as being implicated in, and a possible biomarker of, CVD.

## 14. Conclusion

Initiation of vascular inflammation is a key event in endothelial dysfunction and CVD as a whole. While a small group of markers (ICAM, VCAM, IL-6, cytokines, etc.) have been generally accepted by the scientific community, questions remain regarding the overall biological relevance, as both reporters and effectors of pathophysiological processes.

Epigenetic biomarkers may yield far-reaching prognostic results but, as a technique, is in its infancy. Nevertheless, epigenetic alterations in vasculature have potential to provide novel therapeutic targets for vascular-associated chronic illnesses and initiate novel drug-development pipelines.

Yet, significant challenges remain. Deeper investigation into the physiological roles of MVs* in vivo* is required. A great deal about MVs remains unknown; even their biogenesis is yet to be completely understood. A concerted effort must be made in the direction of MV profiling. Knowledge of miRNA and their associated pathways will prove necessary in order to understand effects on cells and, importantly, unlock their diagnostic and predictive potential. The observation of proinflammatory miRNA upregulation in stimulated MPs suggests that MV miRNA currently presents perhaps the most compelling opportunity for significant progress in the field of vascular research. Platelet miRNA signature may well quantitatively reflect platelet activation* in vivo* and therefore could have huge potential as biomarkers of cardiovascular risk. In addition, correlating platelet physiology and function with its miRNA content may provide new insights into the changes in age and lifestyle-associated epigenetic drift. To this end, it is essential to identify and subsequently interpret miRNA functionality and hence distinguish which of these are most relevant in a clinical setting.

## Figures and Tables

**Table 1:** Microvesicle class based on size, origin, and composition.

Microvesicle type	Size (*μ*m)	Formation	Internal composition	External composition
Exosome	<0.1	Exocytosis	Cytoplasm, protein, miRNA, mRNA	Plasma membrane and surface proteins of parent cell
Microparticle	0.1–1.0	Cell stress/stimuli Cell membrane asymmetry and cytoskeletal deregulation	Cytoplasm, protein, miRNA, mRNA	
Apoptotic body	>1.0	Apoptosis	Cytoplasm, protein, miRNA, mRNA, nuclear fragments	
